# Pancreatic Mass Leading to Left-Sided Portal Hypertension, Causing Bleeding from Isolated Gastric Varices

**DOI:** 10.1155/2014/956490

**Published:** 2014-11-30

**Authors:** Helga Thrainsdottir, Vigdis Petursdottir, Sigurdur Blöndal, Einar S. Björnsson

**Affiliations:** ^1^Section of Gastroenterology, Department of Internal Medicine, The National University Hospital of Iceland, Hringbraut, 101 Reykjavik, Iceland; ^2^Faculty of Medicine, University of Iceland, Vatnsmyrarvegur 16, 101 Reykjavik, Iceland; ^3^Department of Pathology, The National University Hospital of Iceland, Hringbraut, 101 Reykjavik, Iceland; ^4^Department of Surgery, The National University Hospital of Iceland, Hringbraut, 101 Reykjavik, Iceland

## Abstract

Mucinous cystic neoplasms (MCN) are an uncommon form of exocrine neoplasms of the pancreas. Symptoms are most often vague and this makes the diagnosis more difficult. The current case is one of three cases yet reported where the MCN caused left-sided portal hypertension leading to the formation of isolated gastric varices and subsequent bleeding from the varices. In the previously reported cases the main symptom was hematemesis. However in the current case the patient experienced no hematemesis, only isolated incidents of dark coloured diarrhea, but the main symptoms were those of iron-deficiency anemia. We present the case report of a 34-year-old woman who presented with dizziness and lethargy and was found to have 12 cm MCN in the pancreas.

## 1. Introduction

Mucinous cystic neoplasms (MCN) are a rare form of exocrine pancreatic tumors [1]. Even though they are often quite big in size the symptoms are usually vague and they tend to be incidental findings on imaging studies [2]. If the splenic vein is compressed left-sided portal hypertension can pursue [3]. However this situation has only been connected to pancreatic MCN a couple of times before [4, 5]. Left-sided portal hypertension can lead to the formation of isolated gastric varices which can cause life-threatening gastrointestinal bleeding, presenting with melena and hematemesis [6]. Here a case will be reported where the patient presented with symptoms of iron-deficiency anemia after unnoticed blood loss from isolated gastric varices resulting from left-sided portal hypertension caused by pancreatic MCN.

## 2. Case Presentation

A 34-year-old woman presented to the emergency room with a few days history of dizziness and lethargy. Severe anemia was detected (Hb 54 g/L, MCV 79 fl). The patient had not experienced hematemesis or fresh blood in stool but she had experienced an isolated incident of diarrhea, dark in color, two days before presenting to the hospital and at least one time before in the same month. Three weeks earlier she had been admitted to the hospital with the same symptoms and was found to have iron-deficiency anemia. Upper gastrointestinal endoscopy and colonoscopy did not reveal any explanation for the anemia. She was discharged, after receiving a blood transfusion, with a planned capsular endoscopy (CE) but presented to the hospital again with worsening symptoms before the CE was performed.

Her past medical history was unremarkable except for hypothyroidism and her only regular medication was the thyroxin replacement. A year earlier she had suffered a motor vehicle accident with a rather serious frontal collision, but examination at the emergency reception did not indicate need for computerized tomography (CT) scan to be undertaken at the time. She was a nonsmoker and used alcohol in moderate amounts.

**Figure 1:**
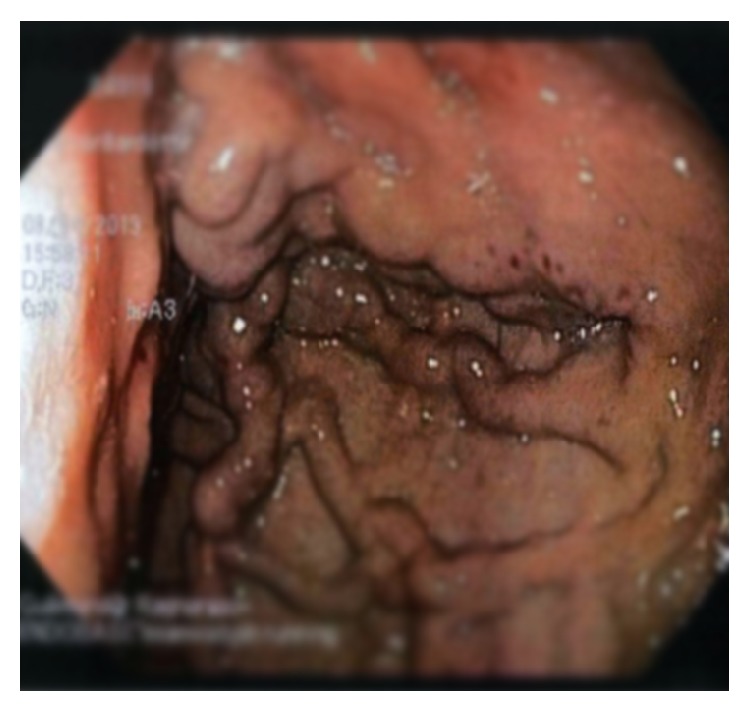
Upper gastrointestinal endoscopy. Isolated gastric varices in the fundus of the stomach and red spots in the mucosa of the varices.

**Figure 2:**
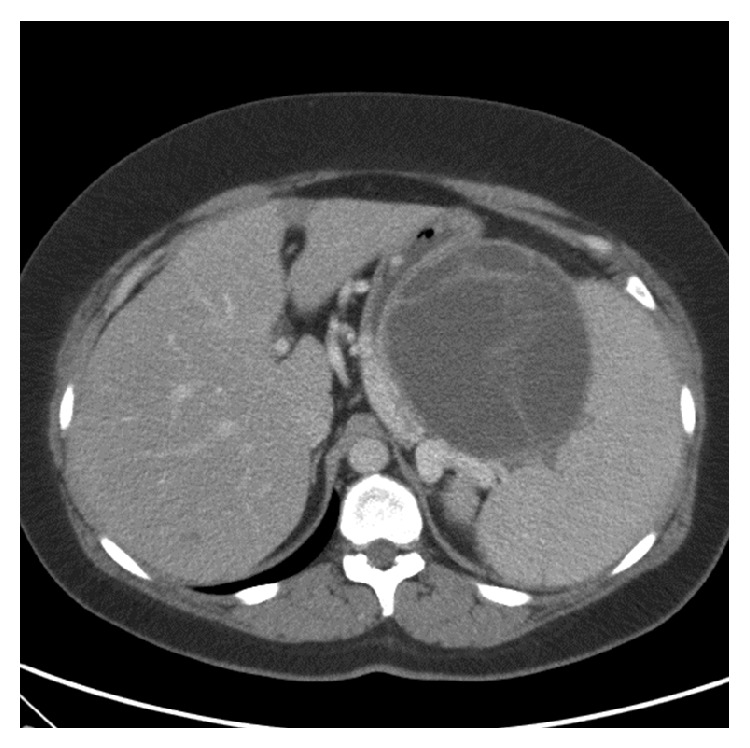
A computed tomography scan of the abdomen. Large cystic tumor in the tail of the pancreas along with moderate splenomegaly.

At the current presentation physical examination revealed tachycardia (126 beats per min) but normal blood pressure (117/66 mmHg) and otherwise normal vital signs. Examination of the abdomen revealed no tenderness, abnormal masses, or organomegalies. Apart from the marked microcytic anemia, other blood tests were within normal range, including liver tests. A slight elevation of the tumor marker CA 19-9 (41.2 U/mL) was found. An upper gastrointestinal endoscopy revealed isolated gastric varices in the fundus of the stomach and red spots in the mucosa of the varices indicating a previous recent bleeding (Figure 1). However no active bleeding was seen and no blood was visible in the stomach. These gastric varices had not been observed in the prior endoscopy, performed three weeks earlier. An abdominal CT was performed in order to identify the underlying pathology, mainly to look for portal vein thrombosis which was the most important differential diagnosis since the patient's liver tests were normal. The images showed a large cystic tumor, 10.4 × 11.4 cm in diameter, located in the tail of the pancreas (Figure 2). Moderate splenomegaly (16 cm) was also detected on the CT.

**Figure 3:**
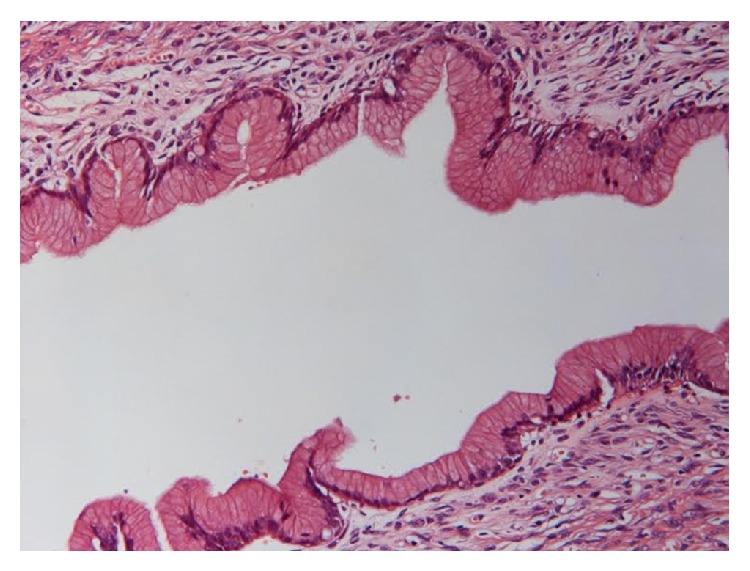
A microscopic picture of the mucinous cystadenoma (expansion ×400). A columnar mucinous epithelium lining cellular stroma.

Laparotomy demonstrated that the tumor had massive adhesions to surrounding organs. In order to resect the tumor a distal pancreatectomy was performed along with a splenectomy, a sleeve resection of the stomach, and a partial hemicolectomy. Macroscopic pathological examination displayed a multilocular cystic tumor, 12 cm in greatest dimension with one large dominating cyst and multiple smaller ones, containing yellowish fluid and no papillary structures on the internal surface. The cyst walls were fibrous and were attached to the surrounding organs. Microscopic examination showed a mucinous, columnar epithelium lining the cystic surfaces, without any cellular or nuclear atypia. The subepithelial stroma was of ovarian-type. These findings were compatible with the final diagnosis of mucinous cystadenoma (Figure 3). Given the previous history of a motor vehicle accident a year earlier, it is conceivable that a traumatic rupture of the cyst has occurred with secondary scar tissue development. The patient had uneventful and full recovery.

## 3. Discussion

Mucinous cystic neoplasms (MCN) of the pancreas are relatively uncommon, comprising about 2–5% of all exocrine neoplasms of the pancreas [1]. They occur almost exclusively in women, most frequently in the fifth decade of life, and are usually situated in the body or tail of the pancreas [7]. MCN are comprised of an inner epithelial layer, rich in mucin secreting cells, supported by an outer layer of dense, ovarial-type stroma. Depending on the atypia in the epithelial layer, they are categorized into adenomas (the most common type, comprising over 70% of cases), borderline tumors, and carcinomas [1, 8]. Being mostly asymptomatic MCN are usually discovered as incidental findings on imaging studies [2]. If any symptoms or signs occur they are vague, such as mild abdominal pain and weight loss, but they can occasionally be acute pancreatitis or a palpable mass. Guidelines suggest that MCN of the pancreas should be surgically removed, because they have the potential to develop into malignancy over time and recurrence hardly ever occurs for noninvasive MCN [2, 7].

Lesions of the body or tail of the pancreas can, in rare situations, cause hindrance to blood flow in the splenic vein and lead to an increased collateral blood flow through the short gastric veins in the fundus of the stomach to the coronary vein and portal vein [3]. This very rare situation, most often caused by pancreatitis, is called left-sided portal hypertension and is characterized by splenomegaly, isolated gastric varices, normal liver tests, and patent extrahepatic portal vein [9]. Isolated fundal varices in the stomach occur in the absence of concomitant esophageal varices and are the least common form of gastric varices [6]. They comprise a risk of upper gastrointestinal bleeding but can be difficult to find on endoscopy since the short gastric veins run subserosally below coarse mucosal folds and can therefore be associated with little mucosal deformity [3, 6].

In spite of having the potential to grow considerably and most often being situated in the body or tail of the pancreas, mucinous cystadenomas are an unusual cause of left-sided portal hypertension and isolated gastric varices. Two cases have been reported before the current case, one from Taiwan [4] and the other from Pakistan [5], but no cases have to our knowledge been reported from other parts of the world. Both of the previous cases describe women (28 and 50 years old, resp.) who presented with vigorous hematemesis, due to bleeding from isolated gastric varices resulting from compression of the splenic vein by MCN of the pancreas [4, 5]. The current case differs from those previously described, due to the fact that the patient did not experience any hematemesis. She experienced isolated incidents of dark diarrhea, but the main symptoms were fatigue and light-headedness due to the iron-deficiency anemia. Furthermore the symptoms developed over a long time, suggesting chronic bleeding from the varices. Iron-deficiency anemia is therefore a rare presentation of MCN of the pancreas. Since the prognosis after resection of the tumor is very good it is important to bear in mind the possibility of pancreatic pathology as an underlying cause of unexplained iron-deficiency anemia.

## References

[1] Hamilton S. R., Aaltonen L. A. (2000). *Pathology and Genetics of Tumours of the Digestive System*.

[2] Zamboni G., Hirabayashi K., Castelli P., Lennon A. M. (2013). Precancerous lesions of the pancreas. *Best Practice and Research: Clinical Gastroenterology*.

[3] Smith T. A., Brand E. J. (2001). Pancreatic cancer presenting as bleeding gastric varices. *Journal of Clinical Gastroenterology*.

[4] Ismail F. W., Mumtaz K., Chawla T., Jafri W. (2007). Gastric variceal bleed in a patient without liver cirrhosis: an unusual cause of haematemesis. *Singapore Medical Journal*.

[5] Tzeng Y.-D. T., Liu S.-I., Tsai C.-C. (2012). An unusual cause of haematemesis: left-sided portal hypertension due to a large pancreatic tumour. *Digestive and Liver Disease*.

[6] Garcia-Pagán J. C., Barrufet M., Cardenas A., Escorsell À. (2014). Management of gastric varices. *Clinical Gastroenterology and Hepatology*.

[7] Farrell J. J., Fernández-del Castillo C. (2013). Pancreatic cystic neoplasms: management and unanswered questions. *Gastroenterology*.

[8] Crippa S., Salvia R., Warshaw A. L. (2008). Mucinous cystic neoplasm of the pancreas is not an aggressive entity: lessons from 163 resected patients. *Annals of Surgery*.

[9] Chang C. Y. (1999). Pancreatic adenocarcinoma presenting as sinistral portal hypertension: an unusual presentation of pancreatic cancer. *Yale Journal of Biology and Medicine*.

